# Supplementary material for: Development of Amino Acids Functionalized SBA-15 for the Improvement of Protein Adsorption
Source: Molecules. 2021 Oct 8;26(19):6085. doi: 10.3390/molecules26196085 (PMC8512485; doi:10.3390/molecules26196085)
Supplement: Supplementary file 1 [file molecules-26-06085-s001.zip › molecules-1405750-supplementary.pdf]

Supplementary data

Development of amino acids functionalized SBA-15 for the improvement of protein adsorption

Raquel Gutiérrez-Climente[1,2,†,*], Margaux Clavié[2,†], Jérémie Gouyon[2], Giang Ngo[3], Yoann Ladner[2], Pascal Etienne[4], Pascal Dumy[2], Pierre Martineau[3], Martine Pugnière[3], Catherine Perrin[2], Gilles Subra[2*], Ahmad Mehdi[1*]

1 ICGM, Univ. Montpellier, CNRS, ENSCM, 34000 Montpellier, France
2 IBMM, Univ. Montpellier, CNRS, ENSCM, 34000 Montpellier, France; margaux.clavie@umontpellier.fr (M.C.); jeremie.gouyon@univ-lorraine.fr (J.G.); yoann.ladner@umontpellier.fr (Y.L.); Pascal.Dumy@enscm.fr (P.D.); catherine.perrin@umontpellier.fr (C.P.)
3 IRCM, Univ. Montpellier, Inserm, ICM, 34000 Montpellier, France; thi-hong-giang.ngo@inserm.fr (G.N.); pierre.martineau@inserm.fr (P.M.); martine.pugniere@inserm.fr (M.P.)
4 I2C, Univ. Montpellier, CNRS, ENSCM, 34000 Montpellier, France; pascal.etienne@umontpellier.fr
* Correspondence: raquel.gutierrez-climente@enscm.fr (R.G.-C.); gilles.subra@umontpellier.fr (G.S.); ahmad.mehdi@umontpellier.fr (A.M.)
† These two authors contributed equally to the work.

Compound 1:

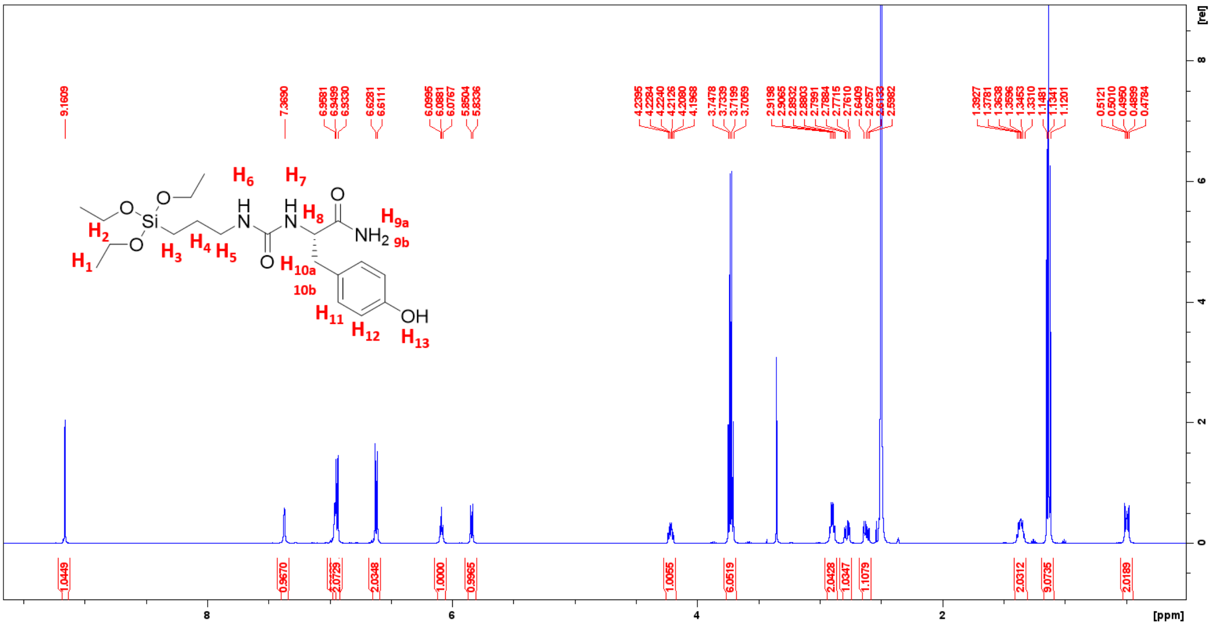

Figure S1. ^1H NMR spectra of silylated tyrosinamide using DIEA as base

^1H NMR (500MHz, DMSO-d6, 25°C): δ(ppm) 0.49 (m, 2H, H_3) ; 1.13 (t, 9H, J = 7.0 Hz, H_1) ; 1.36 (m, 2H, H_4) ; 2.62 (dd, 1H, J = 7.5 Hz, 13.8 Hz, H_{10a}) ; 2.78 (dd, 1H, J = 5.3 Hz, 13.8 Hz, H_{10b}) ; 2.90 (q, 2H, J = 6.4 Hz, H_5) ; 3.73 (q, 6H, J = 7.0 Hz, H_2) ; 4.22 (m, 1H, H_8) ; 5.84 (d, 1H, J = 8.2 Hz, H_7) ; 6.09 (t, 1H, J = 5.4 Hz, H_6) ; 6.62 (d, 2H, J = 8.5 Hz, H_{11}) ; 6.94 (d, 2H, J = 8.4 Hz, H_{12}) ; 6.96 (s, 1H, H_{9a}) ; 7.37 (s, 1H, H_{9b}) ; 9.16 (s, 1H, H_{13}).

Residual DIEA: 0%

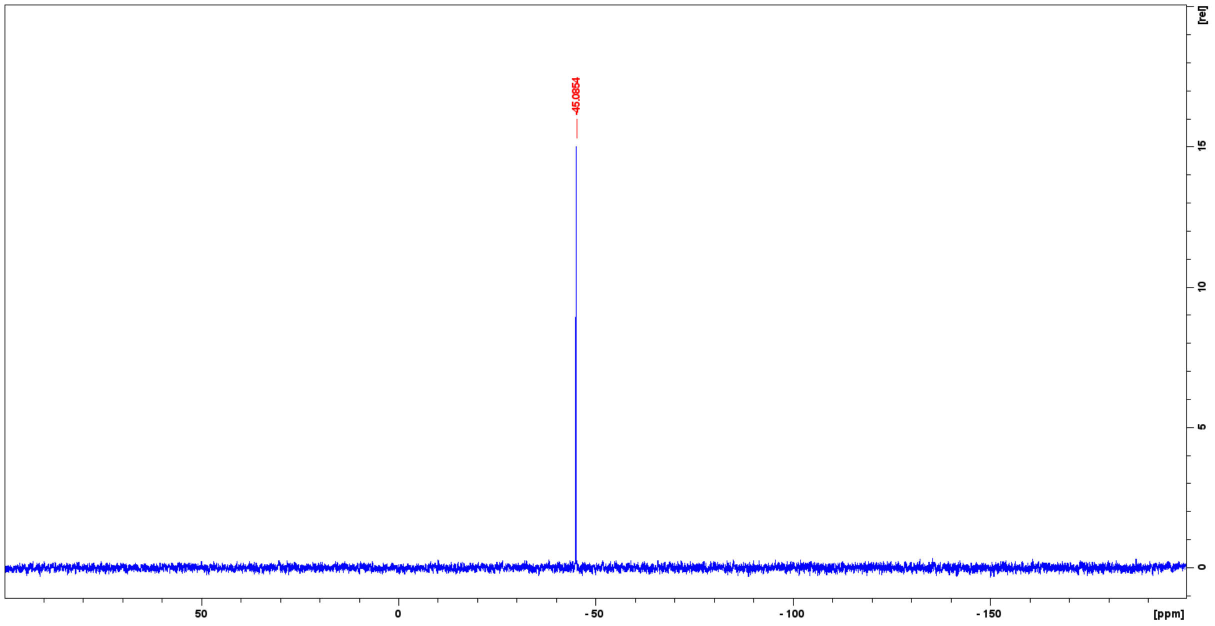

Figure S2. ^{29}Si NMR spectra of silylated tyrosinamide using DIEA as base

^{29}Si NMR (99.4MHz, DMSO-d6, 25°C): δ(ppm) -45.0

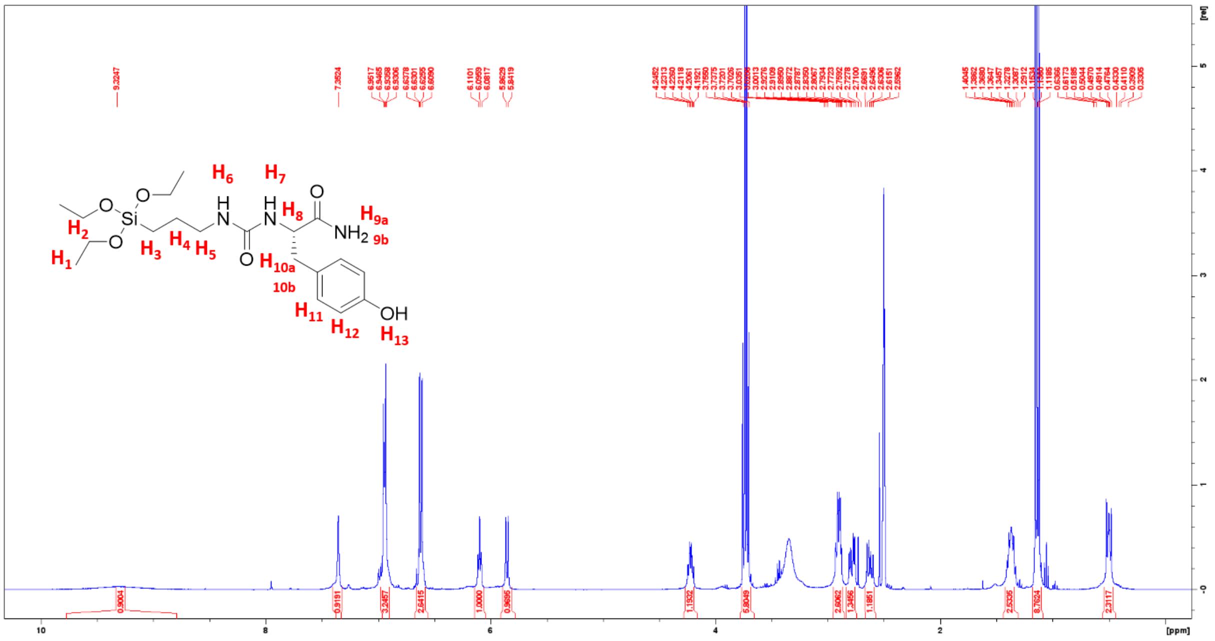

Figure S3. ^{1}H NMR spectra of silylated tyrosinamide using $NaHCO_3$ as base

^{1}H RMN (500MHz, DMSO-d6, 25°C): δ(ppm) 0.49 (m, 2H, H_3) ; 1.13 (t, 9H, J = 7.0 Hz, H_1) ; 1.36 (m, 2H, H_4) ; 2.62 (dd, 1H, J = 7.5 Hz, 13.8 Hz, H_{10a}) ; 2.78 (dd, 1H, J = 5.3 Hz, 13.8 Hz, H_{10b}) ; 2.90 (q, 2H, J = 6.4 Hz, H_5) ; 3.73 (q, 6H, J = 7.0 Hz, H_2) ; 4.22 (m, 1H, H_8) ; 5.84 (d, 1H, J = 8.2 Hz, H_7) ; 6.09 (t, 1H, J = 5.4 Hz, H_6) ; 6.62 (d, 2H, J = 8.5 Hz, H_{11}) ; 6.94 (d, 2H, J = 8.4 Hz, H_{12}) ; 6.96 (s, 1H, H_{9a}) ; 7.35 (s, 1H, H_{9b}).

Compound 2:

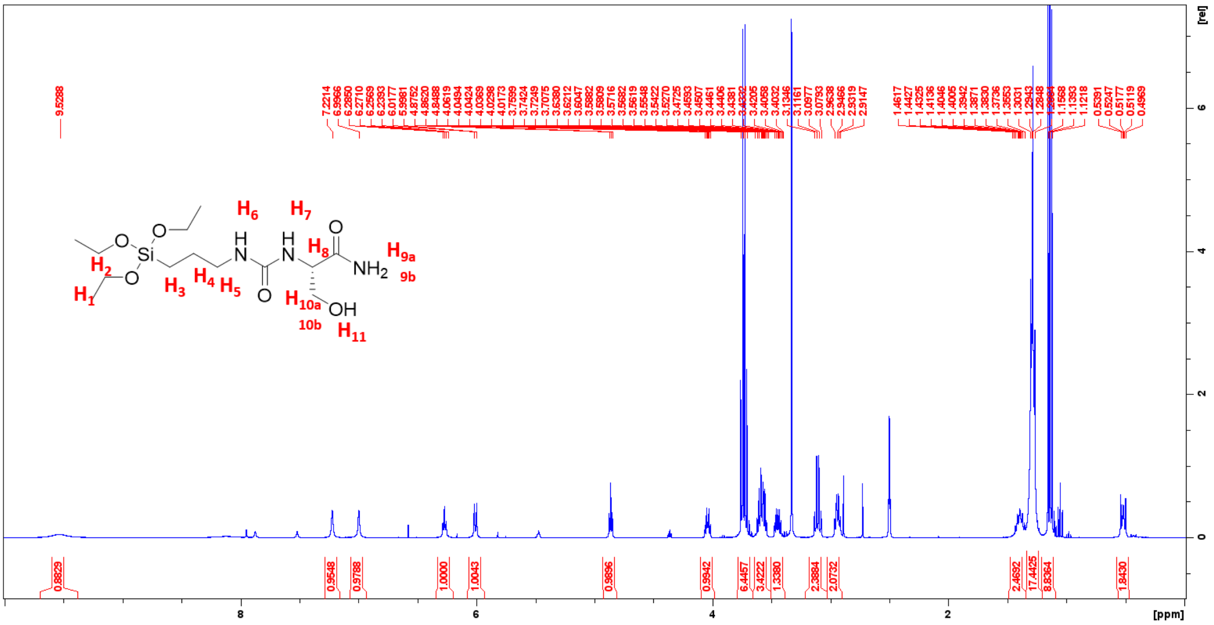

Figure S4. ^{1}H NMR spectra of silylated serinamide using DIEA as base

^{1}H RMN (500MHz, DMSO-d6, 25°C): δ(ppm) 0.52 (m, 2H, H_3) ; 1.14 (t, 9H, J = 7.0 Hz, H_1) ; 1.28 (m, 17H, CH_3 DIEA) ; 1.39 (m, 2H, H_4) ; 2.94 (q, 2H, J = 7.0 Hz, H_5) ; 3.10 (q, 3H, J = 7.5 Hz, CH_2 DIEA) ; 3.45 (m, 1H, H_{10a}) ; 3.58 (m, 3H, H_{10b} + 2CH DIEA) ; 3.73 (q, 6H, J = 7.0 Hz, H_2) ; 4.04 (m, 1H, H_8) ; 4.86 (t, 1H, J = 5.3 Hz, H_{11}) ; 6.00 (d, 1H, J = 7.8 Hz, H_7) ; 6.27 (t, 1H, J = 5.5 Hz, H_6) ; 6.99 (s, 1H, H_{9a}) ; 7.22 (s, 1H, H_{9b}) ; 9.52 (s, 1H, NH^+ DIEA).

Residual DIEA: 25%

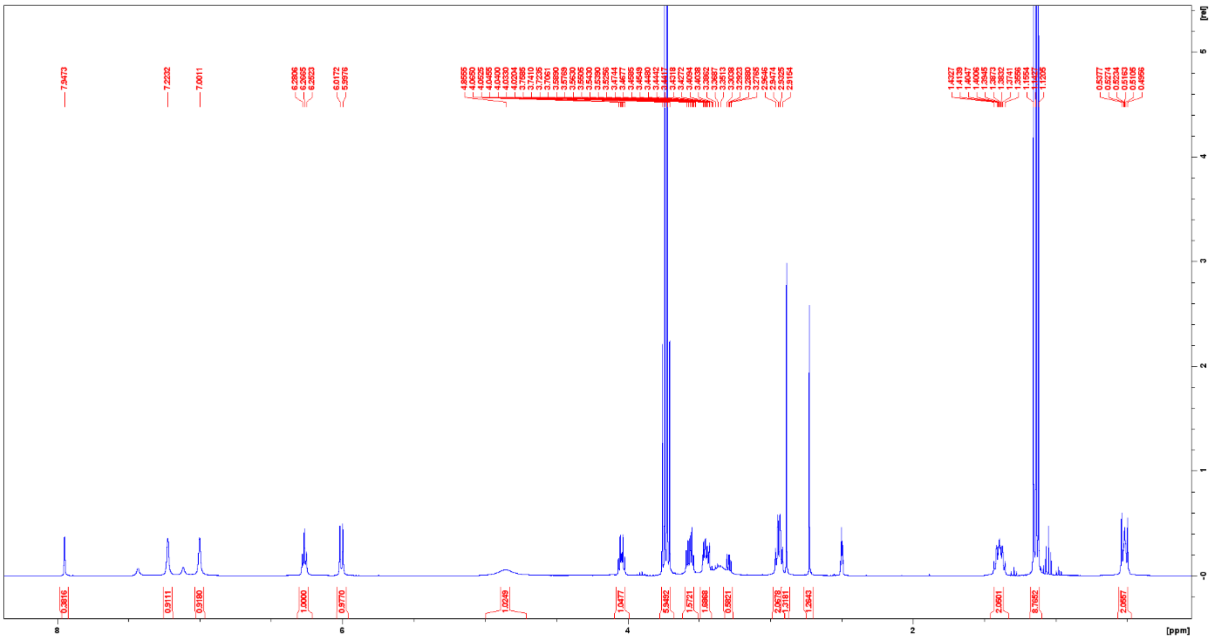

Figure S5. ^{1}H NMR spectra of silylated serinamide using $NaHCO_3$ as base

^{1}H RMN (500MHz, DMSO-d6, 25°C): δ(ppm) 0.52 (m, 2H, H_3) ; 1.14 (t, 9H, J = 7.0 Hz, H_1) ; 1.39 (m, 2H, H_4) ; 2.94 (q, 2H, J = 7.0 Hz, H_5) ; 3.45 (m, 1H, H_{10a}) ; 3.58 (m, 3H, H_{10b} + 2CH DIEA) ; 3.73 (q, 6H, J = 7.0 Hz, H_2) ; 4.04 (m, 1H, H_8) ; 4.86 (s, 1H, H_{11}) ; 6.00 (d, 1H, J = 7.8 Hz, H_7) ; 6.27 (t, 1H, J = 5.5 Hz, H_6) ; 6.99 (s, 1H, H_{9a}) ; 7.22 (s, 1H, H_{9b}).

Compound 3:

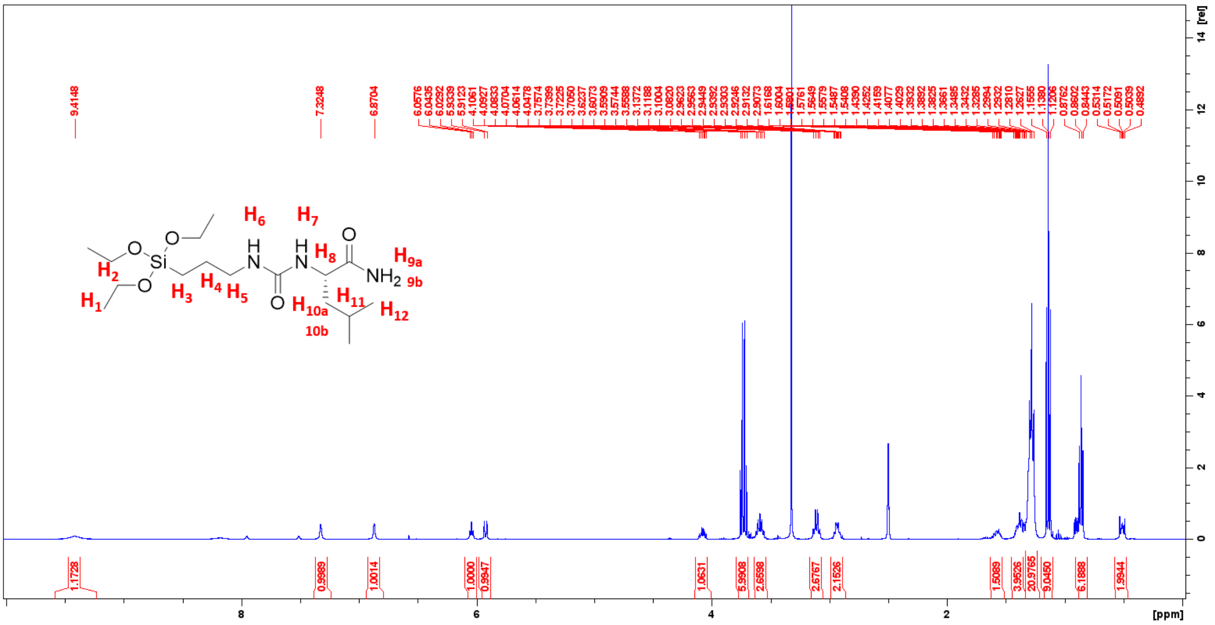

Figure S6. ^{1}H NMR spectra of silylated leucinamide using DIEA as base

^{1}H RMN (500MHz, DMSO-d6, 25°C): δ(ppm) 0.50 (m, 2H, H_3) ; 0.86 (t, 6H, J = 6.5 Hz, H_{12}) ; 1.14 (t, 9H, J = 7.0 Hz, H_1) ; 1.28 (m, 20H, CH_3 DIEA – H_{10a} – H_{10b}) ; 1.38 (m, 2H, H_4) ; 1.57 (m, 1H, H_{11}) ; 2.93 (m, 2H, H_5) ; 3.11 (m, 3H, CH_2 DIEA) ; 3.59 (m, 3H, 2CH DIEA) ; 3.73 (q, 6H, J = 7.0 Hz, H_2) ; 4.07 (m, 1H, H_8) ; 5.92 (d, 1H, J = 8.6 Hz, H_7) ; 6.04 (t, 1H, J = 5.8 Hz, H_6) ; 6.87 (s, 1H, H_{9a}) ; 7.32 (s, 1H, H_{9b}) ; 9.41 (s, 1H, NH^+ DIEA).

Residual DIEA: 57%

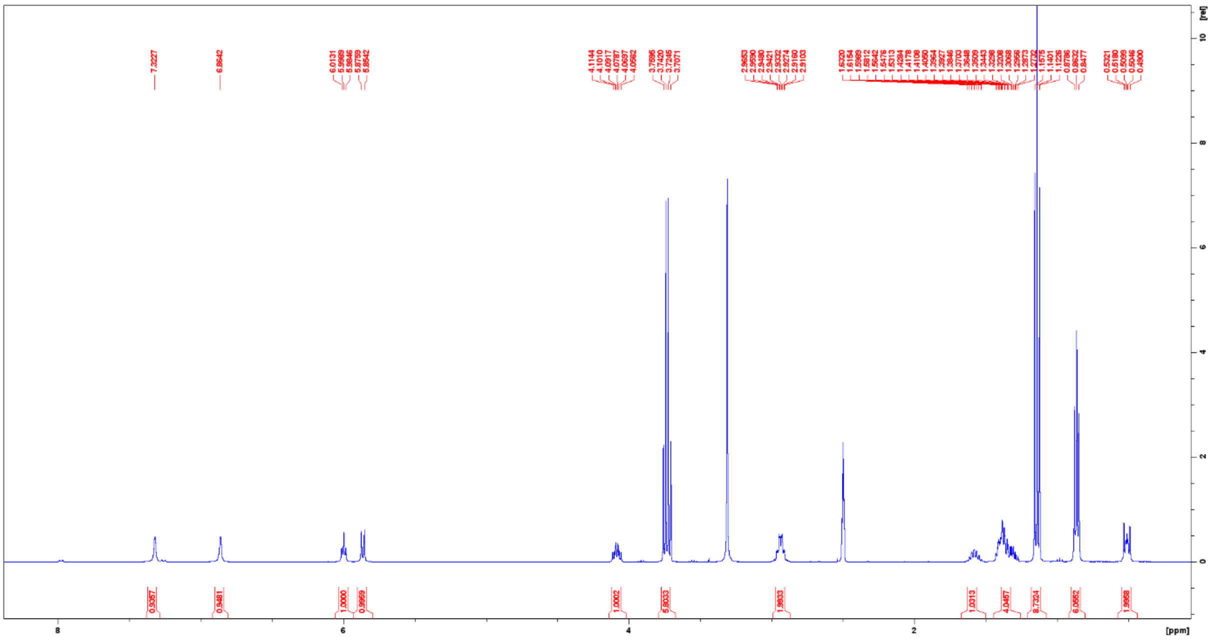

Figure S7. ^{1}H NMR spectra of silylated leucinamide using $NaHCO_3$ as base

^{1}H RMN (500MHz, DMSO-d6, 25°C): δ(ppm) 0.50 (m, 2H, H_3) ; 0.86 (t, 6H, J = 6.5 Hz, H_{12}) ; 1.14 (t, 9H, J = 7.0 Hz, H_1) ; 1.28-1.42 (m, H_4 - H_{10a} – H_{10b}) ; 1.57 (m, 1H, H_{11}) ; 2.93 (m, 2H, H_5) ; 3.73 (q, 6H, J = 7.0 Hz, H_2) ; 4.07 (m, 1H, H_8) ; 5.92 (d, 1H, J = 8.6 Hz, H_7) ; 6.04 (t, 1H, J = 5.8 Hz, H_6) ; 6.87 (s, 1H, H_{9a}) ; 7.32 (s, 1H, H_{9b}).

Compound 4:

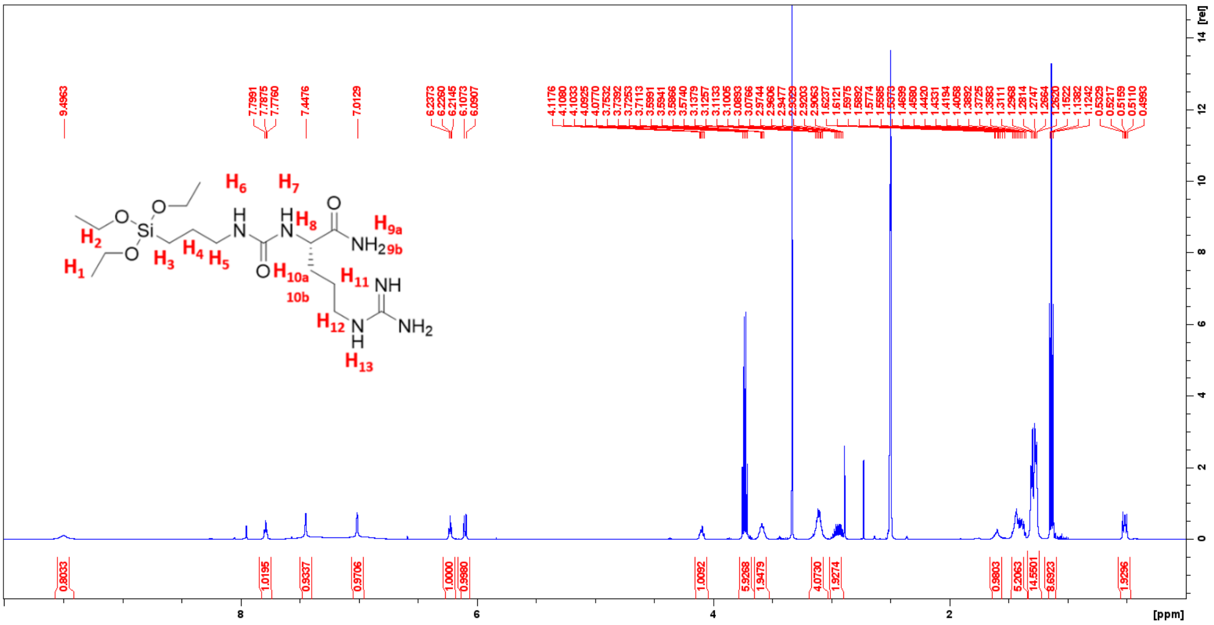

Figure S8. ^{1}H NMR spectra of silylated argininamide using DIEA as base

^{1}H RMN (500MHz, DMSO-d6, 25°C): δ(ppm) 0.52 (m, 2H, H_3) ; 1.14 (t, 9H, J = 7.0 Hz, H_1) ; 1.28 (m, 15H, CH_3 DIEA) ; 1.41 (m, 5H, H_{10a} - H_{10b} - H_4) ; 1.59 (m, 1H, H_{12}) ; 2.94 (m, 2H, H_5) ; 3.10 (m, 4H, CH_2 DIEA – H_{11}) ; 3.59 (m, 2H, 2CH DIEA) ; 3.73 (q, 6H, J = 7.0 Hz, H_2) ; 4.10 (m, 1H, H_8) ; 6.10 (d, 1H, J = 7.8 Hz, H_7) ; 6.22 (t, 1H, J = 5.5 Hz, H_6) ; 7.01 (s, 1H, H_{9a}) ; 7.45 (s, 1H, H_{9b}) ; 7.78 (t, 1H, J = 5.8 Hz, H_{13}) ; 9.49 (s, 1H, NH^+ DIEA).

Residual DIEA: 50%

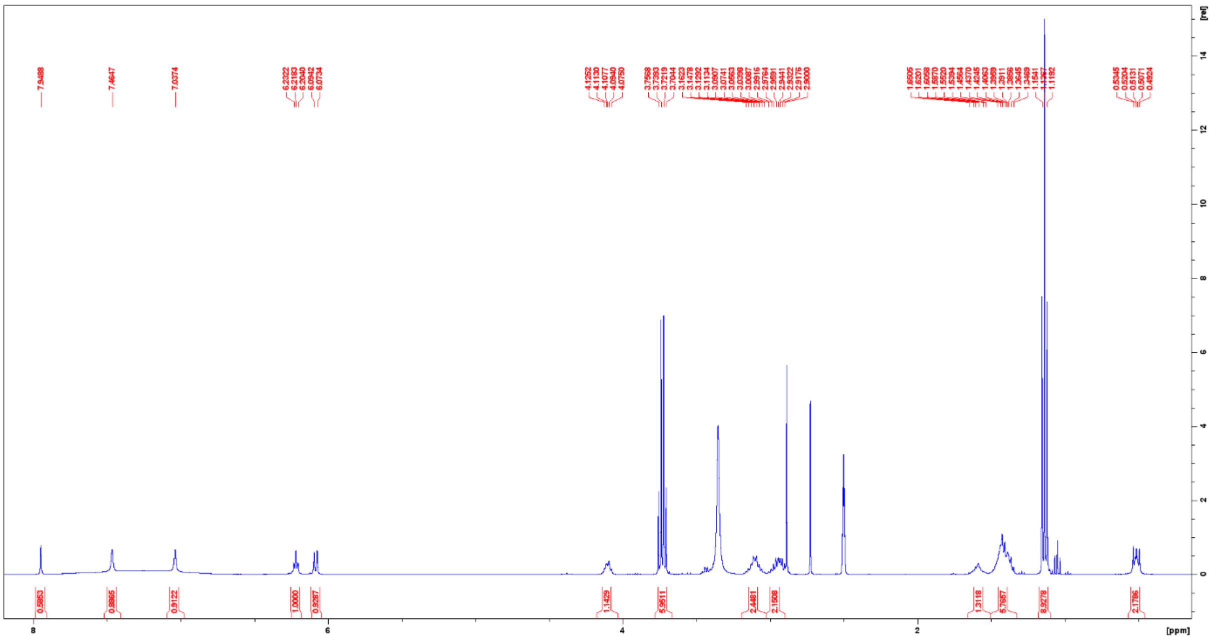

Figure S9. ^{1}H NMR spectra of silylated serinamide using $NaHCO_3$ as base

^{1}H RMN (500MHz, DMSO-d6, 25°C): δ(ppm) 0.52 (m, 2H, H_3) ; 1.14 (t, 9H, J = 7.0 Hz, H_1) ; 1.41 (m, 5H, H_{10a} - H_{10b} - H_4) ; 1.59 (m, 1H, H_{12}) ; 2.94 (m, 2H, H_5) ; 3.10 (m, 2H, H_{11}) ; 3.73 (q, 6H, J = 7.0 Hz, H_2) ; 4.10 (m, 1H, H_8) ; 6.10 (d, 1H, J = 7.8 Hz, H_7) ; 6.22 (t, 1H, J = 5.5 Hz, H_6) ; 7.01 (s, 1H, H_{9a}) ; 7.45 (s, 1H, H_{9b}).

Compound 5:

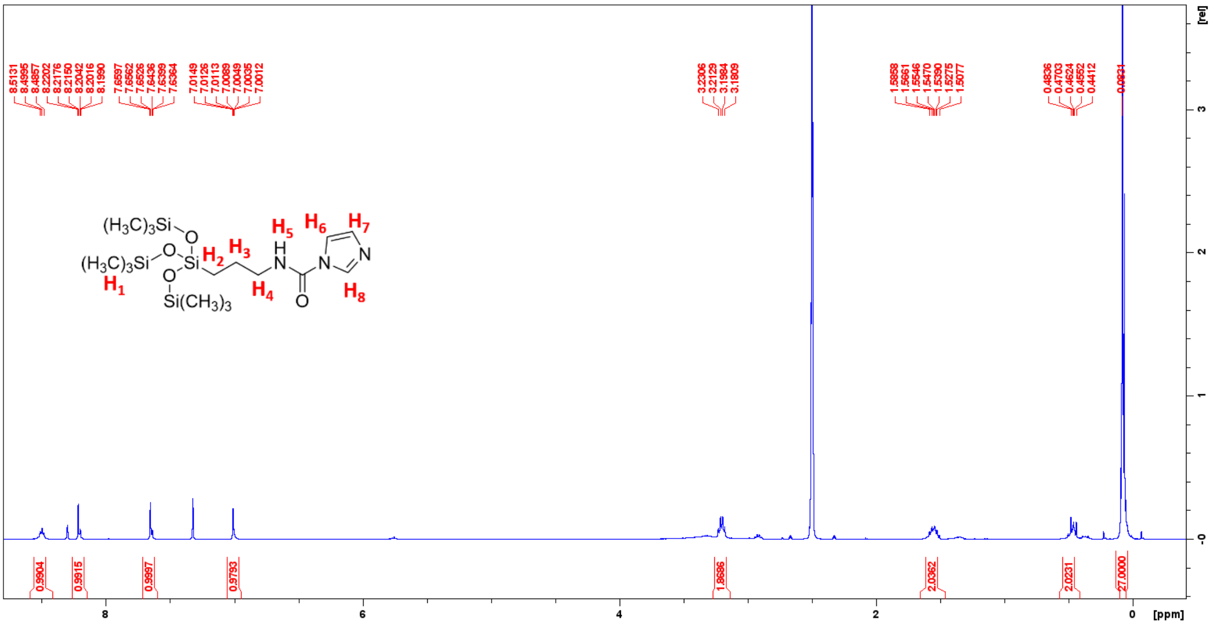

Figure S10. ^{1}H NMR spectra of the synthesis of N-[3-tris(trimethylsiloxy)silylpropyl]-imidazole-1-carboxamide

^{1}H RMN (500MHz, DMSO-d6, 25°C): δ(ppm) 0.08 (s, 27H, H_1) ; 0.46 (m, 2H, H_2) ; 1.55 (3, 2H, H_3) ; 3.20 (q, 2H, J = 5.8 Hz, H_4) ; 7.01 (m, 1H, H_8) ; 7.38 (s, 0.8H, traces of CDI) ; 7.66 (t, 1H, J = 1.5 Hz, H_7) ; 8.21 (t, 1H, J = 1.1 Hz, H_6) ; 8.30 (s, 0.4H, traces of CDI) ; 8.50 (t, 1H, J = 5.5 Hz, H_5).

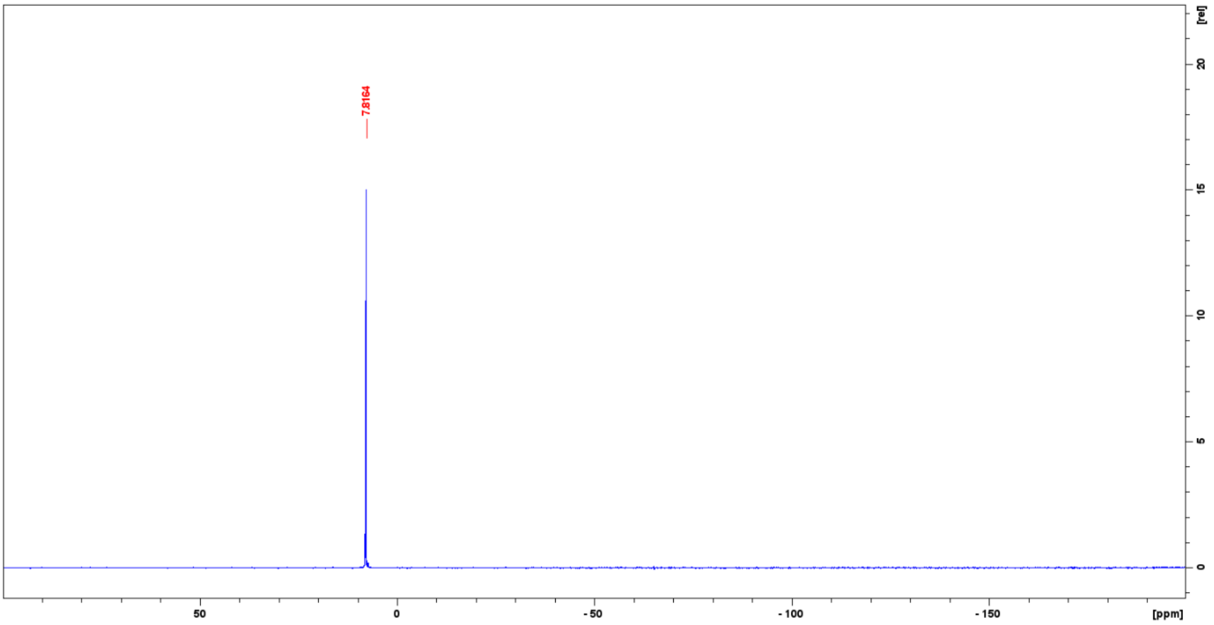

Figure S 11. ^{29}Si NMR spectra of the synthesis of N-[3-tris(trimethylsiloxy)silylpropyl]-imidazole-1-carboxamide

^{29}Si RMN (99.4MHz, DMSO-d6, 25°C): δ(ppm) 7.81

Compound 6:

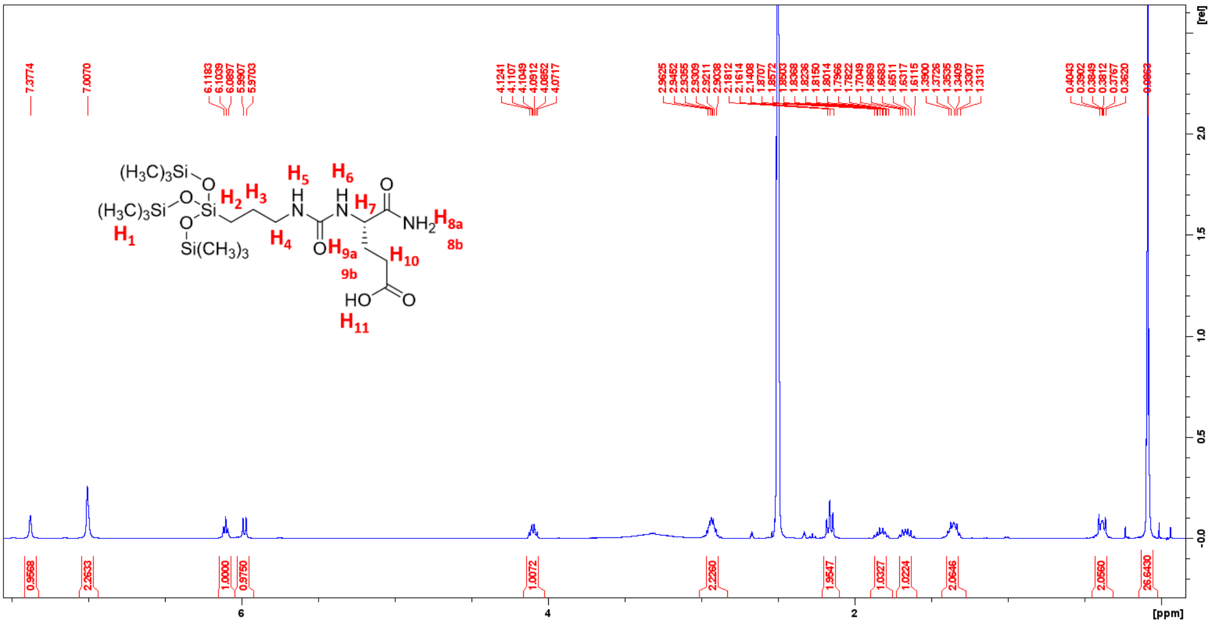

Figure S12. ^{1}H NMR spectra of silylated isoglutamine

^{1}H RMN (500MHz, DMSO-d6, 25°C): δ(ppm) 0.08 (m, 27H, H_1) ; 0.38 (m, 2H, H_2) ; 1.35 (m, 2H, H_3) ; 1.66 (m, 1H, H_{9a}) ; 1.66 (m, 1H, H_{9b}) ; 2.16 (t, 2H, J = 8.0 Hz, H_{10}) ; 2.93 (m, 2H, H_4) ; 4.09 (m, 1H, H_7) ; 5.98 (d, 1H, J = 8.0 Hz, H_6) ; 6.10 (t, 1H, J = 5.6 Hz, H_7) ; 7.00 (s, 2H, H_{8a} – H_{11}) ; 7.37 (s, 1H, H_{8b}).

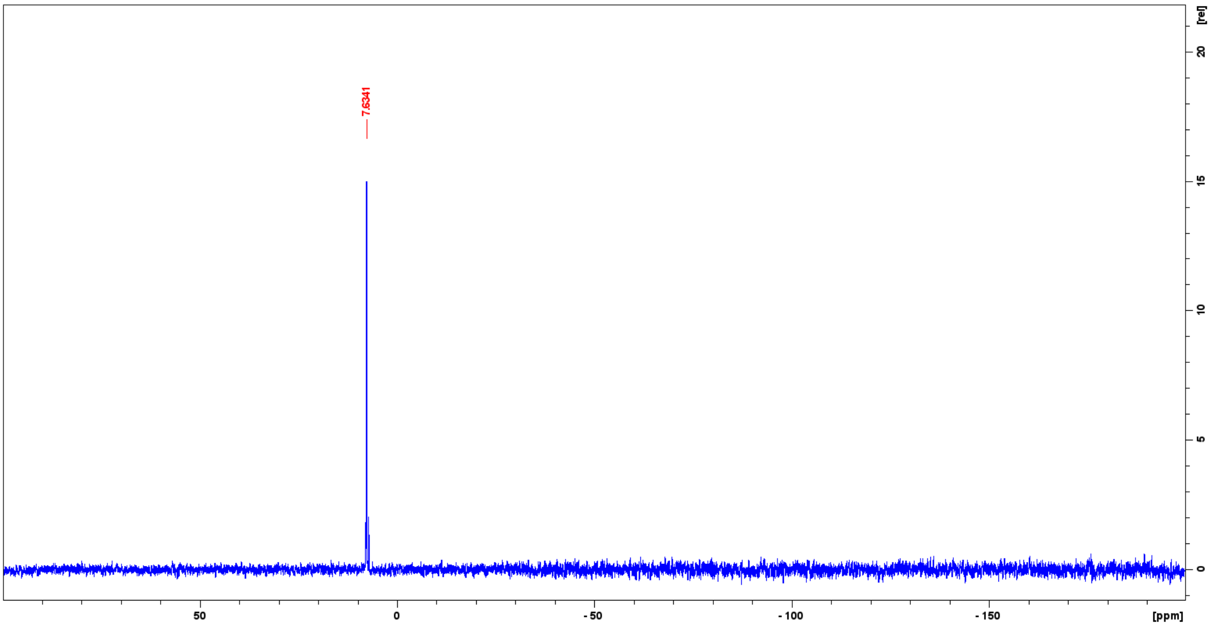

Figure S13. ^{29}Si NMR spectra of silylated isoglutamine

^{29}Si RMN (99.4MHz, DMSO-d6, 25°C): δ(ppm) 7.63
